# Anent the Genomics of Spermatogenesis in *Drosophila melanogaster*

**DOI:** 10.1371/journal.pone.0055915

**Published:** 2013-02-07

**Authors:** Dan L. Lindsley, John Roote, James A. Kennison

**Affiliations:** 1 Department of Cell and Developmental Biology, University of California San Diego, La Jolla, California, United States of America; 2 Department of Genetics, University of Cambridge, Cambridge, United Kingdom; 3 Program on Genomics of Differentiation, Eunice Kennedy Shriver National Institute of Child Health and Human Development, National Institutes of Health, Bethesda, Maryland, United States of America; VIB & Katholieke Universiteit Leuven, Belgium

## Abstract

An appreciable fraction of the *Drosophila melanogaster* genome is dedicated to male fertility. One approach to characterizing this subset of the genome is through the study of male-sterile mutations. We studied the relation between vital and male-fertility genes in three large autosomal regions that were saturated for lethal and male-sterile mutations. The majority of male-sterile mutations affect genes that are exclusively expressed in males. These genes are required only for male fertility, and several mutant alleles of each such gene were encountered. A few male-sterile mutations were alleles of vital genes that are expressed in both males and females. About one-fifth of the genes in *Drosophila melanogaster* show male-specific expression in adults. Although some earlier studies found a paucity of genes on the *X* chromosome showing male-biased expression, we did not find any significant differences between the *X* chromosome and the autosomes either in the relative frequencies of mutations to male sterility or in the frequencies of genes with male-specific expression in adults. Our results suggest that as much as 25% of the Drosophila genome may be dedicated to male fertility.

## Introduction

An appreciable fraction of the Drosophila genome is dedicated to male fertility, and inferentially to the germ line. One approach to characterizing this subset of the genome is through the study of male-sterile mutations. It was noted almost forty years ago that mutations to male sterility in *Drosophila melanogaster* are recovered at frequencies of 10–15% the frequencies of mutations to lethality [1]. This is true for both the *X* chromosome and the autosomes. Does this reflect a large number of genes that are required solely for male fertility, or are many of the male-sterile mutations in genes that are also required for viability and/or for female fertility? Two studies suggested that most genes identified by male-sterile mutations are essential for viability [2], [3]. Both of these studies were done with *X*-linked lethal and male-sterile mutations, which made the determinations of allelism more difficult. It has also been reported that a paucity of genes on the *X* chromosome show male-biased expression [4], [5], [6], making it problematic whether conclusions from sex-linked male-sterile mutations can be extended to the rest of the genome. We took advantage of the availability of a collection of stocks with highly-mutagenized autosomes to revisit this problem. We identified a large number of male-sterile mutations within three genetically characterized regions of the genome and determined which mutations are alleles of genes that can also mutate to lethality and which mutations are alleles of genes that are required only for fertility. We found that the majority of male-sterile mutations in these regions affect fifteen genes that are required only for fertility. Additionally, only three of the fifteen are required in both sexes, with the remaining twelve required only for male fertility. This agrees with earlier observations that the genetic control of gametogenesis is distinct in males and females of Drosophila [7]. We identified the transcription units for eight of the twelve genes that are required only for male fertility. All of these genes show male-specific expression in adults. Surprisingly, almost one quarter of all genes transcribed in adults show male-specific expression. Although few transcripts expressed in adults of both sexes do not correspond to annotated genes, many transcripts with sex-specific expression in adults show no overlap with exons of predicted genes. These sex-specific transcripts appear to identify genes that have not been annotated. The functions of these unannotated genes remain to be elucidated, but these genes appear to account for about 18% of all sex-specific genes. It has been reported that there are fewer genes on the *X* chromosome with male-biased expression [5], [6]. We found that the frequencies of genes with male-specific expression do not appear to differ between the *X* chromosome and the autosomes. In addition, compared to lethal mutations, male-sterile mutations are recovered at about the same frequencies on the *X* chromosome and the autosomes.

## Materials and Methods

An experimental approach to this problem became available with the establishment of the Zuker collection of stocks (∼6000 second chromosome stocks and ∼6000 third chromosome stocks) [8] containing balanced autosomes that had been highly mutagenized by ethyl methane-sulfonate (EMS) (*cn bw/CyO* for chromosome 2 and *bw; st/TM6B*, *Hu Tb* for chromosome 3) and the demonstration that 2396 of these stocks contained male-sterile mutations [9]. The treated autosomes in these lines were originally classified as homozygous viable at their genesis on the basis of survival of *cn bw* or *bw; st* offspring; however, many of the lines were segregating for recessive lethal mutations. The lines segregating for recessive lethal mutations were derived from mosaic gonads [10] and comprised a mixture of two different autosomes, both in balanced condition, one or both of which could carry independently induced lethal mutations. The apparent homozygotes were actually trans-heterozygotes of lethal-bearing chromosomes. The male-sterile mutations in these lines were complete mutations affecting both components of the mosaics. Very often the segregating lethals became fixed in the balanced lines so that homozygous males could no longer be obtained from the stock, although the male-sterile mutations persisted.

In order to examine the allelic relations between the male-sterile and lethal mutations, we selected a subset of male-sterile mutations by screening them against several autosomal deficiencies for regions that had previously been saturated for lethal mutations (Table 1). Males heterozygous for the male-sterile-bearing chromosomes were crossed to females carrying the autosomal deficiencies and the ms/Df progeny tested for male fertility. Accordingly, some of the deficiency heterozygotes failed to survive because the deficiency uncovered an independent lethal mutation that had become fixed in the balanced lines, others were male sterile (locating the male sterile to the deficiency), but the majority were viable and fertile. For those lines in which the mutagenized chromosome heterozygous to the deficiency was either lethal or male sterile, more precise localizations of the mutations were then determined by crossing to a series of included and overlapping deficiencies that subdivided the larger regions into a series of subregions. Mutations falling into particular subregions were then differentiated and allelism determined by complementation tests.

**Table 1:** DNA and essential genes in three autosomal regions.

			Number of essential genes^a^	
Region^b^	Cytology	DNA (kb)	Lethal	Male sterile^c^
Adh	34C1-2;36A6-7	∼2938	54	8
72A-D	71F3-5;72D12	∼315	23	1
76B-D	76B1-2;76D5	∼640	24	6

a totals include the genes identified in this work.

b regions were defined by the deficiencies *Df(2L)b84a7*, *Df(2L)b88c75*, *Df(2L)A48*, and *Df(2L)r10* for Adh, *Df(3L)th102* for 72A-D, and *Df(3L)kto2* for 76B-D.

c includes genes essential only for fertility (male fertility or both male and female fertility). This does not include genes essential for both viability and fertility.

At this point in the analysis we had two collections of mutations for three large autosomal regions. The first collection included the male-sterile mutations mapped to deficiencies and sorted into complementation groups by allelism. The second collection included the lethal mutations similarly mapped and characterized. It remained to determine the complementation relations between them. Several alleles of each male sterile complementation group were crossed to alleles of all of the lethal complementation groups within the same subregion. Males that carried a male-sterile mutation on one chromosome and a lethal mutation on the homolog were examined for viability and fertility.

The molecular natures of several of the male sterile mutations on the third chromosome were determined by amplifying the candidate open reading frames from homozygous or hemizygous males by PCR and sequencing the amplified DNA. The mutant sequence was compared to the sequence of the same candidate gene amplified from the parental *bw*; *st* stock.

For the mutagenesis experiments, we assumed that the recovered mutations followed a Poisson distribution. We calculated the mean number of mutations per chromosome (*m*) from the frequency of mutation-free chromosomes, which equals *e^−m^*. The mean numbers of lethal mutations (*m_l_*), male sterile mutations (*m_ms_*), and female sterile mutations (*m_fs_*) were estimated in this manner.

For the analysis of male-specific transcripts, the high-throughput RNA sequencing (RNA-seq) data from the Developmental Stage Time Course Transcriptional Profiling of the modENCODE Project [11] (http://flybase.org/cgi-bin/gbrowse/dmelrnaseq/) were used to determine the sex-specific adult expression of transcripts in selected regions of the genome. Although many of the genes expressed in adults are also expressed during embryonic, larval, or pupal stages, we did not attempt to determine whether expression at earlier stages of development is sex-specific.

##  Results and Discussion

**Figure 1:**
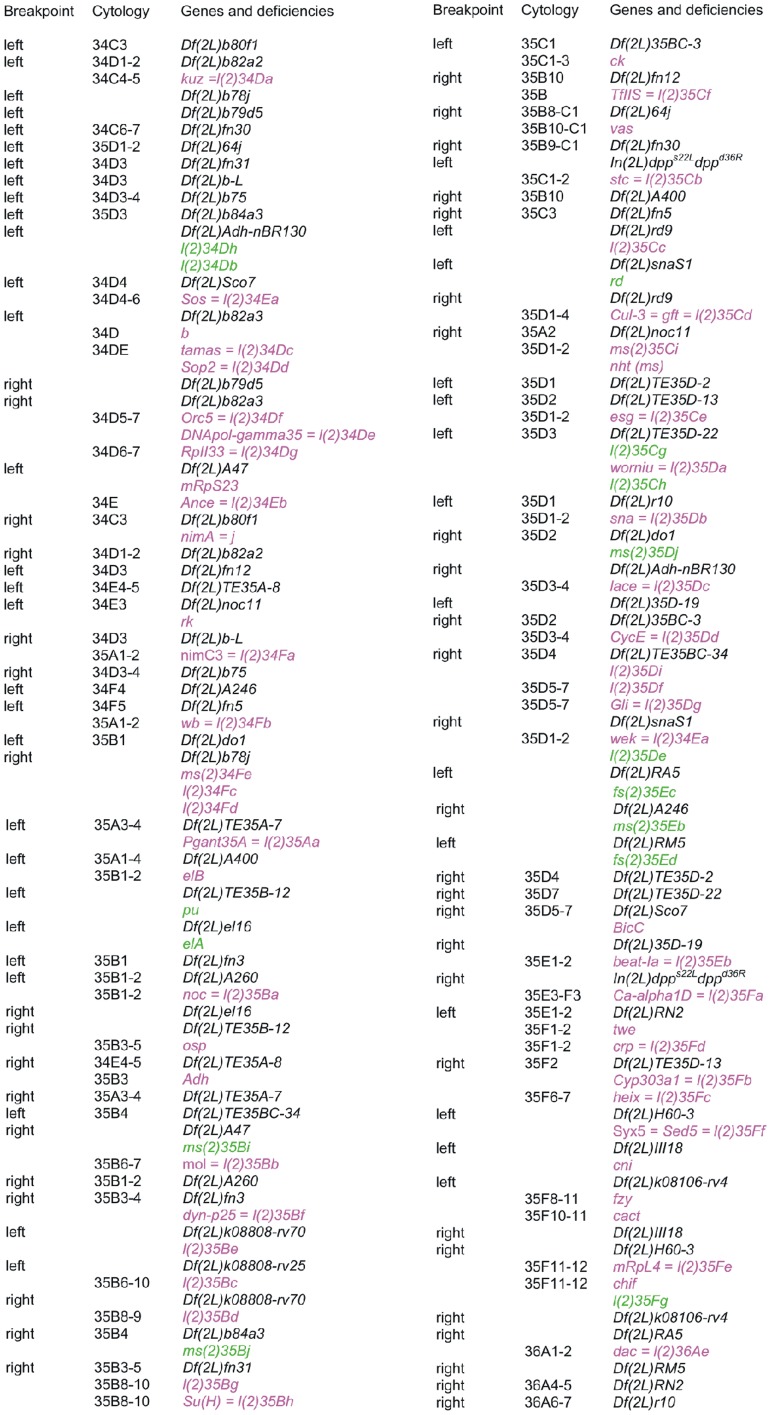
Cytogenetic map showing the deficiencies and genes in polytene chromosome region 34C-36A. The cytological locations are only given for deletions or genes mapped on polytene chromosomes (which do not always correspond to the cytological locations given for the molecular map by the Drosophila Genome Project). Deficiency breakpoints localized on the molecular map are indicated in red and the non-localized breakpoints are indicated in black. Genes are in purple if the transcription unit has been identified, and in green if the transcription unit is not currently known.

**Figure 2:**
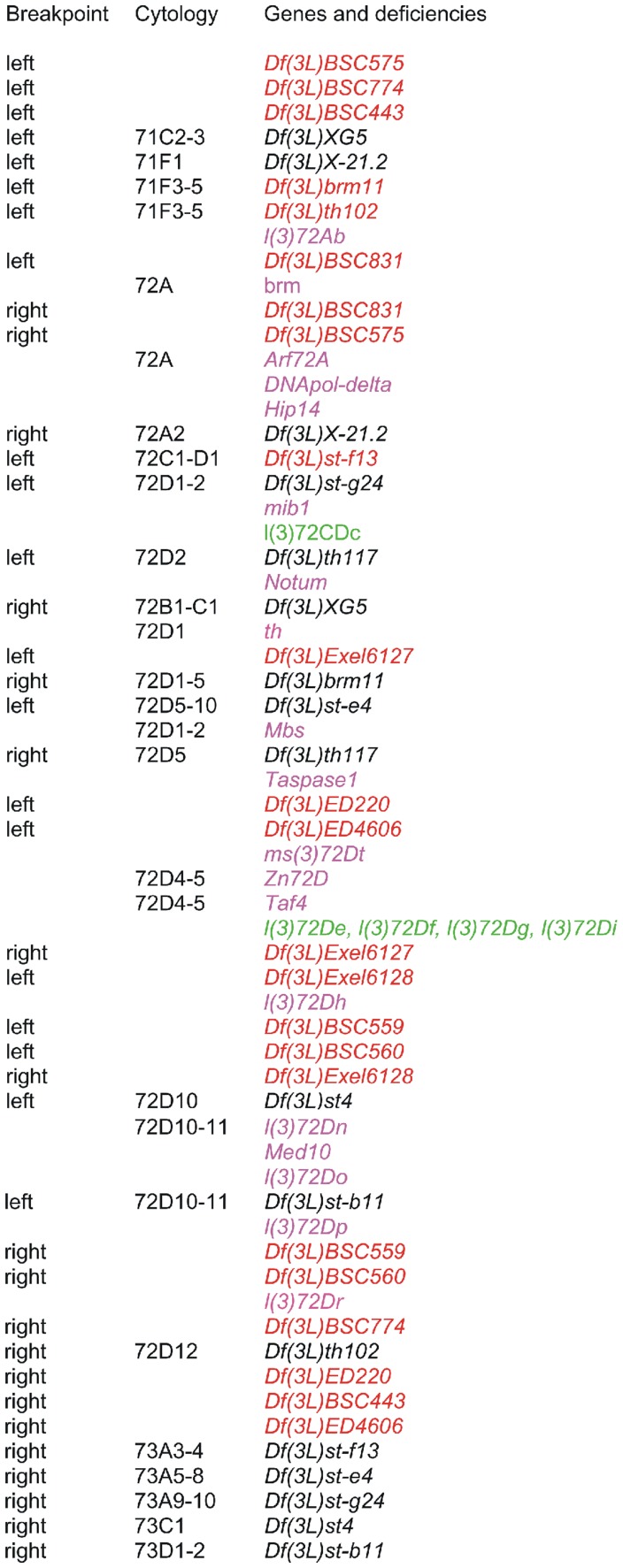
Cytogenetic map showing the deficiencies and genes in polytene chromosome region 72A-D. The cytological locations are only given for deletions or genes mapped on polytene chromosomes (which do not always correspond to the cytological locations given for the molecular map by the Drosophila Genome Project). Deficiency breakpoints localized on the molecular map are indicated in red and the non-localized breakpoints are indicated in black. Genes are in purple if the transcription unit has been identified, and in green if the transcription unit is not currently known.

**Figure 3:**
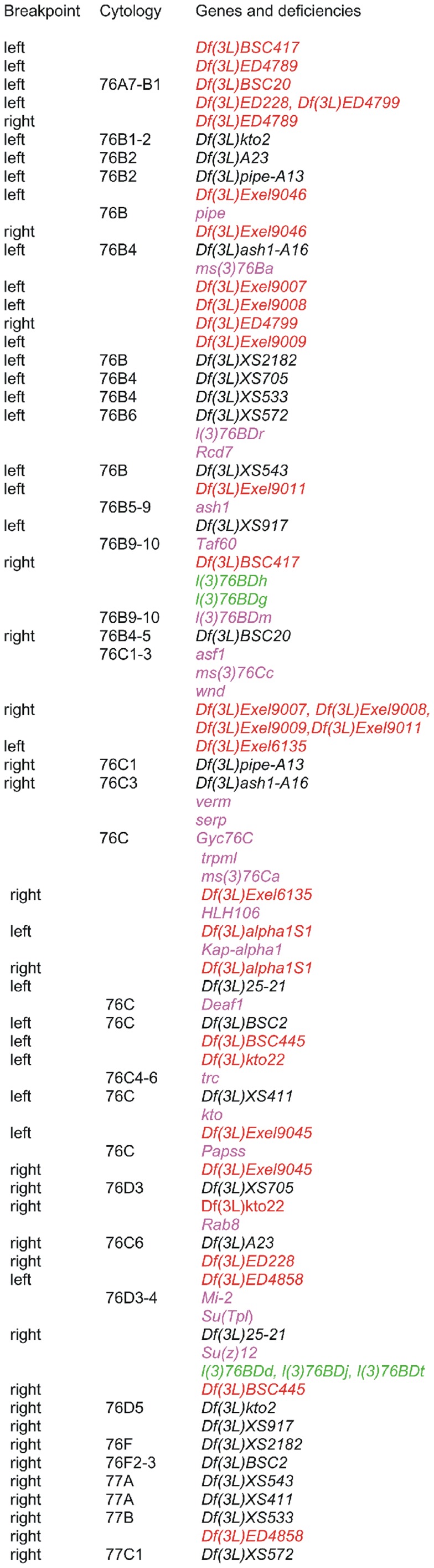
Cytogenetic map showing the deficiencies and genes in polytene chromosome region 76B-D. The cytological locations are only given for deletions or genes mapped on polytene chromosomes (which do not always correspond to the cytological locations given for the molecular map by the Drosophila Genome Project). Deficiency breakpoints localized on the molecular map are indicated in red and the non-localized breakpoints are indicated in black. Genes are in purple if the transcription unit has been identified, and in green if the transcription unit is not currently known.

We previously screened 11,502 lines from the Zuker collection containing autosomes that were initially classified as homozygous viable, and identified 2216 male-sterile lines and 180 barely-fertile lines [9]. We crossed the 968 male-sterile and barely fertile second-chromosome lines to three overlapping deficiencies that span the *Adh* region (∼2938 kb) [12] and identified 48 lines in which the hemizygous males were sterile and four lines in which the hemizygous flies were lethal. We crossed the 1428 male-sterile and barely-fertile third-chromosome lines to both *Df(3L)kto2* (∼640 kb) [13] and *Df(3L)th102* (∼315 kb) [14] and tested hemizygous males for fertility. Forty-one lines were identified in which the hemizygous males were sterile, and sixteen lines in which the hemizygous flies were lethal. When mapping the mutations, we identified one line (*Z0002*) that carried both a lethal mutation and an independent male sterile mutation within *Df(3L)kto2*. The results of the mapping and determination of allelism are summarized in Table 2, Table 3, Table 4, Figure 1, Figure 2, and Figure 3).

**Table 2:** Fertility genes identified by male-sterile mutations from the Zuker collection.

Gene	# of alleles	Adult expression	fertility	Description
*ms(2)34Fe*	11	Male-specific	male sterile	classic^a^ (but many have motile sperm)
*ms(2)35Bi*	15		male sterile, previously predicted ms in *osp* to *l(2)35Bb* interval	nebenkerns vacuolated and misshapen, some nuclear sizes vary
*ms(2)35Bj*	1		male sterile	variable, individualization failure
*nht*	2	Male-specific	male sterile	spermatocyte arrest
*ms(2)35Ci*	1	Male-specific	male sterile	classic^a^; debris along sperm tails
*ms(2)35Dj*	2		male sterile	
*ms(2)35Eb*	1		male sterile, previously-predicted ms in *beat-B* to *BicC* interval	variable, some individualization
*twe*	6	Both sexes	male sterile, some alleles are also female sterile	nebenkerns abnormal; debris
*ms(3)72Dt*	6	Male-specific	male sterile	classic^a^
*ms(3)76Ba*	6	Male-specific	male sterile	nebenkerns large and irregular
*Rcd7*	2	Male-specific	male sterile	dark spheres of nuclear size in early elongating cysts
*ms(3)76Cc*	3	Male-specific	male sterile	classic^a^
*ms(3)76Ca*	5	Male-specific	male sterile	classic^a^
*wnd*	3	Both sexes	male sterile, females are barely fertile	motile sperm, behavioral defects
*Kap-alpha1*	4	Both sexes	male sterile, female fertility variable	individualization failure

a The “classic” male sterile phenotype [9] is a failure during spermatid differentiation, usually with extensive spermatid elongation, little or no sperm individualization and coiling, and the base of the testis filled with debris.

**Table 3:** Male-sterile alleles of vital genes from the Zuker collection.

Gene	# of alleles	Adult expression	fertility	Description
*Cul-3*	6^a^	Both sexes^b^	Male sterile	classic^c^, individualization failure
*dyn-p25*	1	Both sexes	Male sterile	individualization failure
*Gli*	1	Both sexes	Male sterile	classic^c^
*l(2)35Fg*	1		Male sterile	classic^c^
*th*	1	Both sexes	Male sterile	
*l(3)72De*	1		Male sterile	
*Taspase1*	1	Both sexes	Male sterile	
*l(3)76BDg*	5^d^		Male sterile	classic^c^
*Mi-2*	3	Both sexes	Male sterile	spermatocyte arrest
*Hip14*	1	Both sexes	Semi-lethal and male sterile	
*l(3)72Dh*	1	Both sexes	Semi-lethal and male sterile	

a Allele *Z1062* complements *Z1812*, but both fail to complement *Z1089*.

b *Cul-3* appears to have a male-specific promoter.

c The “classic” male sterile phenotype [9] is a failure during spermatid differentiation, usually with extensive spermatid elongation, little or no sperm individualization and coiling, and the base of the testis filled with debris.

d Alleles *Z1128* and *Z6059* fail to complement each other, but complement *Z3146*.

**Table 4:** Lethal alleles of vital genes from the Zuker collection.

Gene	# of alleles	Male fertile when heterozygous to hypomorphic alleles^b^	Lethal when heterozygous to hypomorphic alleles^b^
*l(2)34Dh* a	1		
*wb*	1		
*ck*	1		
*lace*	1		
*l(3)72Ab*	4	*Z5080, Z1604, Z4474*	*Z2850*
*Hip14*	1		*Z4772*
*l(3)72De*	1		
*Taspase1*	2	*Z1087, Z1560*	
*l(3)72Dp*	1		
*ash1*	2	*Z1369, Z6088*	
*Taf6*	1		
*HLH106*	1	*Z6151*	
*Papss*	1		
*Rab8*	1		
*Mi-2*	1		

a New lethal locus in *kuz*-*l(2)34Db* interval.

b The hypomorphic alleles tested were *l(3)72Ab^4^*, *l(3)72Ab^16^*, *Hip14^72Ad-1^*, *Hip14^72Ad-2^*, *Taspase1^1^*, *Taspase1^2^*, *ash1^B3^*, *HLH106^3^*, *HLH106^5^*, *HLH106^6^*, *HLH106^7^*.

### Most male-sterile mutations are not alleles of genes essential for viability

We believe that the lethal mutations that we identified in our deficiency tests came from mosaic gonads and are independent of the recessive male sterility of the original lines. The alternative hypothesis is that they are haplo-specific lethals [15] that are viable but male sterile when homozygous. Such haplo-specific lethal mutations should survive and be male sterile when homozygous or when heterozygous to other hypomorphic alleles of the same gene. To test this alternative hypothesis, we examined the 16 third chromosome lines that were lethal when heterozygous to a deficiency. Although homozygous mutant males were recovered for the initial screening in every line [9], homozygous flies are no longer found in any of the 16 lines. We were also able to test 10 of the 16 mutations for complementation with previously identified hypomorphic alleles (Table 4). For two mutations, no males (or females) were recovered in combination with hypomorphic alleles. For the other eight mutations, males heterozygous for the lethal mutations identified in this study and previously identified hypomorphic alleles survived, but were fertile. These results are consistent with the proposition that the lethal mutations came from mosaic gonads and are independent of the male-sterile mutations.

Three-quarters of the male-sterile mutations (68/90) are in 15 genes that appear to be required only for fertility, and not for zygotic viability. Only 22 of the 90 male sterile mutations (24%) are alleles of eleven essential genes. While a few of the male-sterile alleles of essential genes show reductions in viability and/or female fertility, most are male sterile when heterozygous to lethal alleles, but show little or no decrease in viability. That the male sterile alleles of essential genes are special alleles is also suggested by their frequency. Although we recovered an average of almost 5 alleles for each of the genes required only for fertility, we recovered an average of only 2 alleles each for the genes also essential for zygotic viability. For the three genomic regions that we have characterized, there are at least 101 genes essential for zygotic viability and an additional 15 genes required for male fertility (Table 1 and Table 2). Of the 15 genes required for male fertility, 4 {*ms(2)34Fe, ms(3)72Dt*, *Rcd7*, and *ms(3)76Cc*} are in the sperm proteome [16].

### Most male-sterile mutations do not affect female fertility

Of the 15 fertility genes that we identified, 12 are male-specific. The transcription units for three of the male-specific fertility genes that had been previously identified {*ms(2)34Fe*, *nht*, and *ms(2)35Ci*} all appear to be expressed in adult males, but not adult females (Table 2). Based on these results, we sequenced candidate transcription units with male-specific expression from mutants of five of the other male-specific fertility genes. We were able to identify the transcription units for all five (Table 5). Three of the fertility genes (*twe*, *wnd*, and *Kap-alpha1*) are required for both male and female fertility, although not all alleles are female sterile. We also identified the mutant lesions in the *wnd* and *Kap-alpha1* alleles from the Zuker collection (Table 5). As expected, the three genes that are required for both male and female fertility are expressed in both adult males and adult females (Table 2). Of the male-sterile mutations that we tested, about 12% were also female sterile. This is consistent with the earlier estimate that about 9% of autosomal male-sterile mutations also affect female fertility [7].

**Table 5:** Amino-acid polymorphisms and mutations associated with male-sterile mutations from the Zuker collection.

Gene	CG	Allele	Polymorphisms^a^	Mutation^b^
*ms(3)72Dt*	*CG5389*	*Z0797*	T10K	A358T
*ms(3)72Dt*	*CG5389*	*Z1317*	T10K	L366F
*ms(3)76Ba*	*CG14087*	*Z3367*	T453P	W301@
*ms(3)76Ba*	*CG14087*	*Z3156*	T453P	34 bp deletion
*Rcd7*	*CG14098*	*Z0002*		W312@
*Rcd7*	*CG14098*	*Z2196*		R232@
*ms(3)76Cc*	*CG9392*	*Z1193*	S550N, P622S, P851S, D1027G	P402S
*ms(3)76Cc*	*CG9392*	*Z4217*	S550N, P622S, P851S, D1027G	D472N
*ms(3)76Cc*	*CG9392*	*Z5464*	S550N, P622S, P851S, D1027G	Y522N
*wnd*	*CG8789*	*Z2013*	A770T	V327E
*wnd*	*CG8789*	*Z2269*	A770T	Q535@
*wnd*	*CG8789*	*Z5800*	A770T	Q255@
*ms(3)76Ca*	*CG14101*	*Z0796*		A43T
*ms(3)76Ca*	*CG14101*	*Z2365*		N69D
*Kap-alpha1*	*CG8548*	*Z1703*		Q343@
*Kap-alpha1*	*CG8548*	*Z4826*		34 bp deletion
*Kap-alpha1*	*CG8548*	*Z5120*		11 bp deletion+3 bp insertion
*Kap-alpha1*	*CG8548*	*Z5234*		W202@

a Polymorphisms present in the *bw; st* strain before mutagenesis and also present in the mutant alleles.

b@ represents a stop codon.

### Male-sterile mutations are more frequent than female-sterile mutations

We have compared the frequencies of male-sterile mutations, female-sterile mutations, and lethal mutations from published experiments (Table 6). We have only included experiments where the frequencies of two different classes of mutations were reported. In the experiments that examined both male-sterile and female-sterile mutations, male-sterile mutations were recovered at 1.5 to 1.9 times the frequency of female-sterile mutations. Male-sterile mutations were recovered at 11–22% the frequency of lethal mutations, while female-sterile mutations were recovered at 5–10% the frequency of lethal mutations.

**Table 6:** Relative frequencies of lethal and sterile mutations after EMS mutagenesis.

							Ratios		
Chromosome (reference)	Lethal/Total	*m_l_*	Male sterile/Total	*m_ms_*	Female sterile/Total	*m_fs_*	*m_ms_*/*m_l_*	*m_fs_*/*m_l_*	*m_ms_*/*m_fs_*
*X* [1]	1360/4442	0.365	140/3060	0.047			0.13		
*X* [26]	74/199	0.465	8/125	0.066			0.14		
*X* [27]	60%	0.916			95/1064	0.094		0.10	
*X* [28]	66%	1.079			320/5524	0.059		0.05	
*2* [1]	134/270	0.686	4/52	0.080			0.12		
*2* [8]	31865/37944	1.833	970/5398	0.198			0.11		
*2* [29]	11431/18782	0.938			529/7351	0.075		0.008	
*2* [7]			31/376	0.086	24/423	0.058			1.5
*3* [1]	175/270	1.045	8/39	0.230			0.22		
*3* [8]	28329/34586	1.710	1254/6104	0.230			0.13		
*3* [7]			226/2122	0.113	144/2454	0.060			1.9

For most experiments, the numbers of chromosomes that carry lethal or sterile mutations/the total number of chromosomes tested were reported. For two of the *X* chromosome samples, the percentage of the chromosomes carrying lethal mutations was estimated from the sex ratio. *m_l_*, *m_ms_*, and *m_fs_* are the mean numbers of lethal, male sterile, and female sterile mutations per chromosome, respectively.

### Male-sterile mutations are as frequent on the *X* chromosome as on the autosomes

In the genus Drosophila, there is a paucity of genes on the *X* chromosome showing male-biased expression [4], [5], [6], [17], suggesting that the *X* chromosome is a disfavored location for genes selectively expressed in males. For example, Joslyn used a differential cDNA screen from hand-dissected testes to isolate clones of genomic DNA that included only genes expressed specifically in the adult male reproductive organs; he examined expression patterns and polytene locations of the sequences so isolated. Of 56 sequences expressed in the germ line, identified by their failure to be expressed in the germ-lineless sons of *tud* mothers, only 3 mapped to the *X* chromosome [4]. Since he sampled highly expressed genes, he concluded “that genes that are relatively highly expressed in male germ cells are underrepresented on the *X* chromosome, whereas spermatogenic genes, in general, are evenly distributed”. This conclusion is supported by RNAseq annotation 5.32 in which the most highly expressed 10% of testis-expressing genes include 20 *X*-linked:204 autosomal genes, with total expressions of *ca* 16,000 to 260,000 respectively. Parisi and colleagues used competitive hybridization to DNA microarrays of 14,142 predicted *D. melanogaster* transcripts and found that 14% to 17% of autosomal transcripts showed male-biased expression in adults while only 10% of sex-linked transcripts showed male-biased expression [5]. However, the paucity of *X*-linked genes with male-biased expression is not universal among diptera. Genes with male-biased expression are not under-represented on the *X* chromosome of the mosquito *Anopheles gambiae* [18]. Given the reported paucity of genes with male-biased expression on the *X* chromosome of *D. melanogaster*, we were surprised that there does not appear to be a corresponding difference in the frequencies of EMS-induced male-sterile mutations between the *X* chromosome and the autosomes (Table 6). Male-sterile mutations were recovered at about 12% the frequency of recovery of lethal mutations for both the *X* chromosome and both major autosomes. How do we reconcile these disparate observations?

### A large proportion of genes show male-specific expression in adults

The recent availability of high-throughput RNA sequencing (RNA-seq) data from the Developmental Stage Time Course Transcriptional Profiling of the modENCODE Project [11] (http://flybase.org/cgi-bin/gbrowse/dmelrnaseq/) allowed us to re-examine the finding that the *X* chromosome has a paucity of genes showing male-biased expression, but shows no corresponding reduction in the frequency of male-sterile mutations recovered after mutagenesis. We began by examining whether transcription in adult flies is sex-specific for each of the nineteen molecularly-identified genes for which we recovered male-sterile mutations (Table 2). The eight genes that are required only for male fertility are expressed in adult males, but not adult females. The three genes that are required for fertility of both sexes and the eight genes that are essential for viability are all expressed in both sexes in adult flies. We next chose three autosomal regions (∼4.5% of the autosomes) that overlapped the regions that we screened for male sterile-mutations. We also chose two *X* chromosomal regions (∼24% of the *X* chromosome) that overlapped the regions previously screened for male-sterile mutations [2], [3]. We then examined the modENCODE Project data for each region to determine which genes are expressed in adults and whether the adult expression is sex-specific (Table 7). For both the *X* chromosome and the autosomes, we estimate that about 20% of the annotated genes expressed in adults are male specific. The frequency of genes with female-specific expression in adults is at least an order of magnitude lower. We also found many sex-specific transcripts in adults that do not correspond to exons of genes annotated in Release 5.26 of the *D. melanogaster* genome sequence. These sex-specific adult transcripts appear to identify substantial numbers of genes with male-specific expression that have not yet been annotated. From the data in Table 7, we estimate that about 18% of sex-specific genes were not annotated (39 of the 214 sex-specific genes in the genomic regions that we examined have not been annotated). This proportion is the same for the *X* chromosome and the autosomes. When we include genes that have not yet been annotated in our analysis, there is still no significant difference between the *X* chromosome and the autosomes in the frequencies of genes with male-specific adult expression. If there is no paucity of genes with male-specific expression on the *X* chromosome, what is the significance of the paucity of genes with male-biased expression? It was found that among insertions of a transposon containing a testis-specific reporter gene, the average expression levels of the reporter gene from insertions on the *X* chromosome was only about one-quarter the average expression levels from insertions on the autosomes [19], [20]. Recent work suggests that lower expression levels in the testis may be a property of all *X*-linked genes [21]. Why is this significant? Lower average expression levels for genes on the *X* chromosome would result in fewer genes showing male-biased expression above the arbitrary thresholds set in previous analyses [4], [5], [6], [7], [17]. Two recent publications [22], [23] have reexamined the paucity of male-biased genes on the *X* chromosome of Drosophila. Both groups concluded that there does not appear to be a paucity of testis-biased genes on the *X* chromosome. Since we expect that the great majority of male-specific genes will be testis specific, it is not surprising that we find no paucity of male-specific genes on the *X* chromosome.

**Table 7:** Genes with sex-specific expression in adults.

				Genes with adult expression					
				Total		Male-specific		Female-specific	
Polytene region	DNA coordinates^a^	kbp	Genes examined	A^b^	N^c^	A^b^	N^c^	A^b^	N^c^
9B1;11E8	9980k–13157k	3177	*CG34104* to *comt*	304	8	52	6	6	2
19D1;20F4	20299k–22432k	2124	*CG11710* to *CG13865*	106	15	28	9	0	2
34C3;36A2	13500k–16488k	2988	*B4* to *dac*	226	11	63	11	5	0
71F;72E1	15918k–16311k	393	*Pka-C3* to *CG13062*	67	4	5	3	0	1
75F2-4;76E1	19000k–20000k	1000	*nkd* to *CG7668*	113	5	16	5	0	0
*X*-linked total		5301		410	23	80	15	6	4
Autosomal total		4401		406	20	84	19	5	1

a DNA coordinates from Release 5.27.

b A are annotated transcription units.

c N are novel transcription units that have not been annotated.

### Most male-specific genes are not paralogs of genes expressed in both sexes

The observation that 20–25% of genes are expressed only in males is striking. This high proportion is even more notable when we consider that only 1–2% of genes are female-specific. Why are there so many genes that appear to function only in males? It has been noted previously that there are testis-specific paralogs for many subunits of some large protein complexes in Drosophila. For example, five of the thirteen TBP-associated factors (TAFs) have testis-specific paralogs [24]. Testis-specific paralogs have also been described for 12 of the 33 proteasome subunits [25]. Could most of the male-specific genes be paralogs of genes that are expressed in both sexes? For the 164 male-specific genes that we had identified (Table 7), we searched for paralogous proteins with at least 25% identity. We identified paralogs for 65 of these 164 genes. Many genes had multiple paralogs. Forty-one of the male-specific genes have one or more paralogs that are also male-specific. Only 40 of the 164 male-specific genes have at least one paralog that is expressed in both sexes. Thus, the majority of genes with male-specific expression are not simply sex-specific paralogs, but encode functions unique to males.
